# The relationship between metabolic syndrome and increased risk of Barrett’s esophagus: an updated systematic review and meta-analysis

**DOI:** 10.1186/s12876-020-01267-2

**Published:** 2020-05-06

**Authors:** Mohammad Karimian, Majid Salamati, Milad Azami

**Affiliations:** 1 Department of General Surgery, Faculty of Medicine, Ilam University of Medical Sciences, Ilam, Iran; 2 Faculty of Medicine, Ilam University of Medical Sciences, Ilam, Iran

**Keywords:** Metabolic syndrome, Barrett’s esophagus, Meta-analysis

## Abstract

**Background:**

The relationship between metabolic syndrome (MetS) and Barrett’s esophagus (BE) is still a challenging issue, and inconsistent results have been reported in different studies. Therefore, this study was conducted to determine the relationship between MetS and BE.

**Methods:**

In this study, we followed the MOOSE protocol and results were reported according to the PRISMA guidelines. All study steps were performed independently by two authors. If necessary, the dispute was resolved by consultation with a third author. The search strategy is designed to find published studies. Comprehensive search was done in the following databases until July 2019: Cochrane Library, PubMed/Medline, Web of Science, Science Direct, EMBASE, Scopus, CINAHL, EBSCO, and Google Scholar search engine. All analyses were performed using Comprehensive Meta-Analysis Software Ver.2, while *p*-value lower than 0.05 was considered significant.

**Results:**

In 14 studies with a sample size of 108,416, MetS significantly increased the risk of BE (OR = 1.354; 95% CI: 1.145–1.600; *P* < 0.001; Heterogeneity: I^2^ = 81.95%; *P* < 0.001). Sensitivity analysis by omitting one study showed that overall estimates are still robust. Subgroup analysis was significant for continent (*P* < 0.001) and MetS diagnostic criteria (*P* = 0.043), but was not significant for variables of study type (*P* = 0.899), study setting (*P* = 0.115), control groups (*P* = 0.671) and quality of studies (*P* = 0.603). The Begg (*P* = 0.912) and Egger’s (*P* = 0.094) tests were not significant; therefore, the publication bias did not play a role in the results.

**Conclusion:**

MetS increases the risk of BE compared to control groups. The results of this study can help health practitioners by identifying a treatable risk factor for the most important risk factor for esophageal carcinoma (ie, BE). Future studies should examine whether treatment for MetS reduces the risk of BE.

## Background

Barrett’s esophagus (BE) is often defined as a change in any length of the epithelium of the esophagus that can be diagnosed as columnar-type mucosa in endoscopy and is confirmed as intestinal metaplasia through esophageal biopsy [1]. BE is considered a precancerous condition that is closely related to esophageal cancer, especially esophageal adenocarcinoma (EAC) [2]. The prevalence of BE and the incidence of EAC has increased in Western countries [3]. The most important risk factor for BE is gastroesophageal reflux disease (GERD), while other risk factors include male gender, hiatus hernia, and smoking [4, 5].

Metabolic syndrome (MetS) is a complex disorder that includes central obesity, hypertension (HTN), hyperglycemia, hypertriglyceridemia and high-density lipoprotein cholesterol (HDL-C). In addition to being related to cardiovascular disease, diabetes, and polycystic ovary syndrome, MetS and its elements are also linked with various gastrointestinal diseases and abnormal liver function [6, 7]. This disease affects one-fifth of the population in developed countries and its incidence increases with age. The prevalence of MetS is approximately 24% in the United States, 12% in Europe and 10–40% in most Asian countries [8, 9].

Based on recent evidence, the prevalence of both diseases is increasing rapidly. Hence, the relationship between MetS and BE has been hypothesized [8, 9]. Most studies investigating the relationship between obesity and BE have shown that obesity can lead to a significant increase in the risk of BE [4, 5].

Although numerous studies have shown that any of the MetS criteria (i.e., abdominal obesity, hyperglycemia, and HTN) can be a risk factor for BE, the relationship between MetS and BE is still a challenging issue, and inconsistent results have been reported in different studies [10–20]. Meta-analysis is a subset of systematic reviews. The systematic review seeks to collect empirical evidence that meets the predicted eligibility criteria to answer a specific research question. Meta-analysis results may include a more accurate estimate of the impact of treatment or risk factors for disease or other outcomes by combining individual studies [21, 22]. Therefore, this study was conducted to determine the relationship between MetS and BE.

## Method

### Study protocol

In this study, we followed the Meta-analyses Of Observational Studies in Epidemiology (MOOSE) [21–23] protocol and results were reported according to the Preferred Reporting Items for Systematic Reviews and Meta-analysis (PRISMA) (S1 file) [24] guidelines. All study steps were performed independently by two authors. If necessary, the dispute was resolved by consultation with a third author.

### Search strategy

The search strategy is designed to find published studies. Comprehensive search was done in the following databases until July 2019:

Cochrane Library (Cochrane Database of Systematic Reviews - CDSR), PubMed/Medline, Web of Science (ISI), Science Direct, EMBASE, Scopus, CINAHL, EBSCO, and Google Scholar search engine.

There were no restrictions based on language or release date. The search was done using the following MeSH keywords: “Metabolic Syndrome”[Mesh], “Gastroesophageal Reflux”[Mesh], “Esophagitis”[Mesh], “Barrett Esophagus”[Mesh], and “Esophagus”[Mesh].

Combined search in PubMed was done as follows: (((“Metabolic Syndrome”[Mesh]) AND “Gastroesophageal Reflux”[Mesh]) OR (“Esophagus”[Mesh] OR “Barrett Esophagus”[Mesh])) OR “Esophagitis”[Mesh]. Reference lists were screened from all relevant studies to find potential articles.

### Study selection

Two authors (M.A, M.K, or M.S) screened the titles and abstracts independently and then reviewed the full text of the retrieved studies for eligibility based on the defined criteria. If necessary, the dispute was resolved by consultation with a third author (M.A).

### Inclusion and exclusion criteria

This study included prospective and retrospective studies (e.g. cohort, case-control and cross-sectional studies). The language of the published articles was considered in all languages and no historical restrictions were placed on the search. Google Translate and a relevant language teacher were referred to for the translation of non-English texts if necessary. The exclusion criteria were: duplicate studies, studies that did not differentiate BE from GERD, being irrelevant; low quality in qualitative assessment; case studies, review articles, letters to the editor without quantitative data and theses.

### Data extraction

If available, the following data were extracted according to the aim of the study: first author’s name, year of publication, year of review, country/continent, information about the study population (specific groups, population size for the entire sample, case, control, male and female in each case and control groups), number of BE positive patients in each case and control, study design, setting, adjusted or unadjusted odds ratio (OR_s_) or relative risk (RR_s_), diagnostic criteria for MetS, and quality assessment score.

### Quality assessment

Methodological quality was assessed using the Newcastle-Ottawa Quality Assessment Scale [25] for both cohort or case-control studies based on study design and its adapted type for cross-sectional studies. The scale is based on three categories: 1. sample selection (4 points), 2. Comparability of groups (2 points), and 3. Level of exposure/outcome (3 points). Therefore, a maximum of 9 points can be attained. The different levels of methodological quality were defined as follows: 0–5 points: low quality, 6–7 points: average quality, and 8–9 points: high quality.

### Statistical analysis

We combined the studies with the odds ratio (OR_s_) index and 95% confidence interval. In studies that did not report OR_s_ and 95% confidence intervals, we calculated them based on the total sample size of each group as well as the number of MetS positive cases in each of the case (BE) and control (Non-BE) groups. I^2^ index and Q test were used to evaluate the heterogeneity of studies. A *P* value below 0.10 in the Q test for heterogeneity is considered as significant. Cut-off points for I^2^ were defined as 0–24%, 25–49%, 50–74%, and 75–100% for low, medium, high and very high, respectively [26, 27]. According to significant heterogeneity, we used random effects model in meta-analysis. We performed sensitivity analysis for the stability of pooled estimation through omission of only one study. To find out the cause of the heterogeneity, we performed subgroup analysis based on study type (cohort, case-control and cross-sectional), setting (hospital-based and population-based), control groups (BE baseline, colonoscopy, with reflux symptoms, without reflux symptoms, endoscopy), MetS diagnostic criteria (IDF [International Diabetes Federation], WHO [World Health Organization] and NCEP ATP III [National Cholesterol Education Program Adult Treatment Panel III]), quality of study (medium quality and high quality) , and continent (America, Asia, Europe and Oceania). Meta-regression analysis was also performed based on the year of publication. Funnel plot and Begg and Egger’s tests were used to assess publication bias [28, 29]. All analyses were performed using Comprehensive Meta-Analysis Software Ver.2, while *p*-value lower than 0.05 was considered statistically significant.

## Results

### Search results and study characteristics

**Fig. 1:**
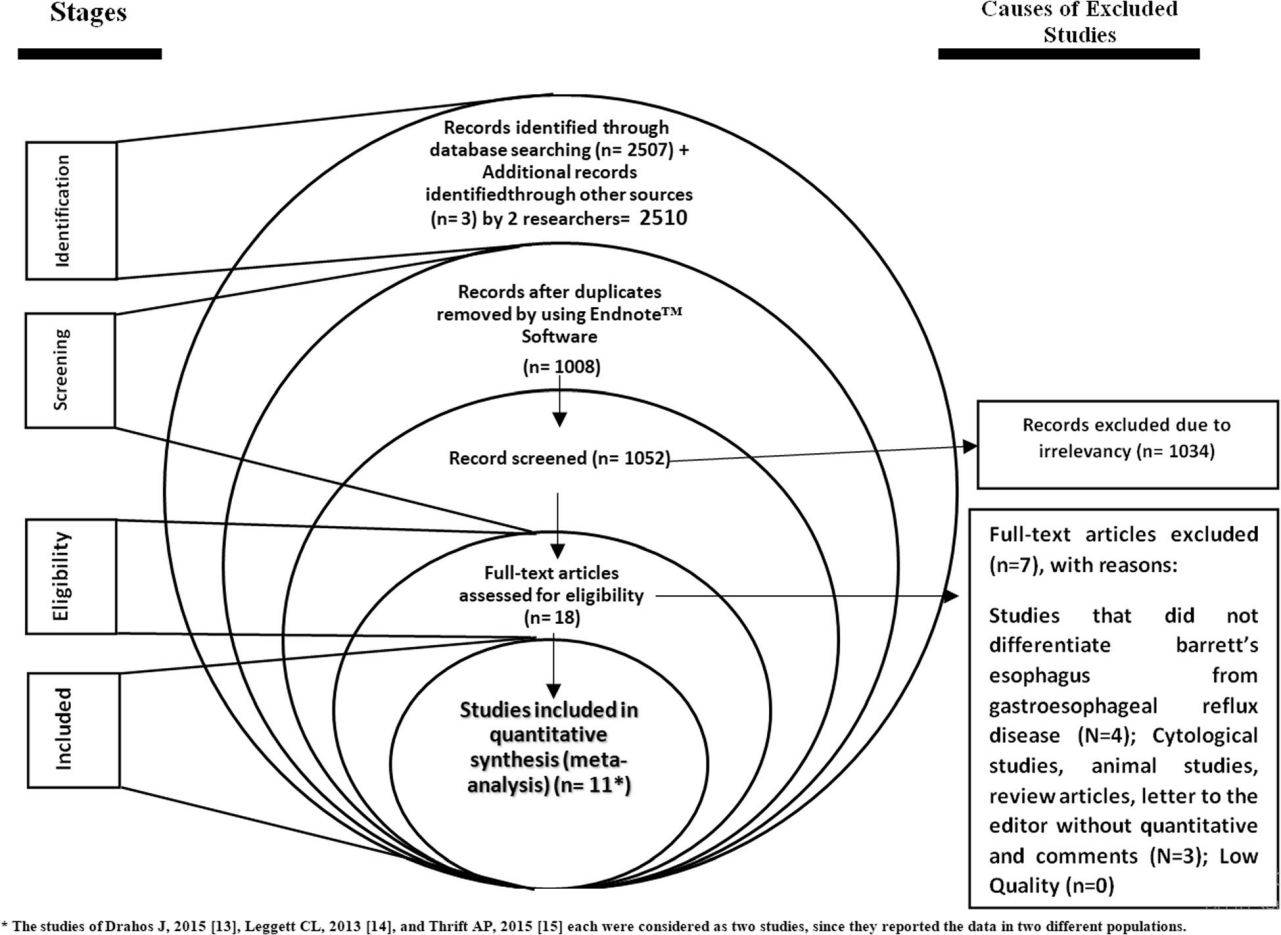
The studies selection process for meta-analysis

The electronic search has identified 2510 studies. A total of 2492 studies were excluded based on the review of title and abstract. Another 7 articles were excluded because they did not meet our inclusion criteria. Eleven articles met our inclusion criteria and were included in the meta-analysis (the studies of Drahos J, 2015 [13], Leggett CL, 2013 [14], and Thrift AP, 2015 [15] each were considered as two studies, since they reported the data in two different populations) (Fig. 1). The characteristics of the studies are shown in Table 1.

**Table 1:** Summary of characteristics in studies into a meta-analysis

Ref.	First author, Published Year	Design	Year of study	Place	Study setting	Special groups	Controls groups	criteria for MetS	Sample size					Effect size			QS
									All	Case		Control		OR	95% CI		
										All	M,F	All	M,F				
[10]	Lee SW, 2017	Cross-sectional	2006–9	Taiwan	Population_based		Endoscopy control	IDF	6594	95	61:34	6499	3447:3052	2.7	1.97	3.71	8
[11]	Healy L.A, 2010	Case-control	2003	Ireland	Hospital_based	–	Control with reflux symptoms	IDF	231	118	79: 39	113	67: 46	1.2	0.707	2.037	8
[12]	Wani SB, 2008	Case-control	NR	USA	Hospital_based	93.2% Caucasians	Control with reflux symptoms	Not mentioned	309	103		206		0.659	0.406	1.068	7
[13]	Duggan C, 2013	Cohort	1995–2009	USA	Hospital_based	96.4% White	BE baseline	IDF	388					1.14	0.56	2.36	7
[14]	Drahos J, 2015	Cross-sectional	2009	USA	Population_based	87.4% White	Endoscopy control	NCEP-ATP III	8792	2198	1150:1048	6594	3450:3144	1.2	1.07	1.36	9
[14]	Drahos J, 2015	Cross-sectional	2009	USA	Population_based	85.3% White	Endoscopy control	NCEP-ATP III	8170	2198	1150:1048	5972	3036:2936	0.93	0.83	1.04	9
[15]	Leggett CL, 2013	Case-control	1999–2006	USA	Population_based	96.4% Caucasians	Control without reflux symptoms	IDF and WHO	206	103	70:33	103	70:33	1.9	1.03	3.6	8
[15]	Leggett CL, 2013	Case-control	1999–2006	USA	Population_based	96.4% Caucasians	Control with reflux symptoms	IDF and WHO	206	103	70:33	103	70:33	2.0	1.1	3.65	8
[16]	Thrift AP, 2015	Case-control	2008–2011	USA	Hospital_based	100% White man	Colonoscopy control	NCEP-ATP III	453	244		209		1.67	1.1	2.55	8
[16]	Thrift AP, 2015	Case-control	2008–2011	USA	Hospital_based	100% White man	Endoscopy control	NCEP-ATP III	859	244		615		0.87	0.49	1.54	8
[17]	Drahos J, 2016	Cross-sectional	1992–2012	United Kingdom	Population_based		Endoscopy control	NCEP-ATP III	60,382	10,215	6399:3816	50,167	31,375:18792	1.12	1.0	1.25	8
[18]	Wu P-C, 2019	Cross-sectional	2016–2018	South Korea	Population_based		Endoscopy control	IDF	4943	88	66:22	4855	2475:2380	2.07	1.29	3.33	9
[19]	Drahos J, 2017	Cross-sectional	2003–2009	USA	Population_based		Endoscopy control	NCEP-ATP III	16,410	575		15,835		1.318	1.098	1.583	9
[20]	Kendall B, 2010	Case-control	2003–6	Australia	Population_based		Endoscopy control	Not mentioned	473	236		237		1.91	1.32	2.76	6

*MetS* Metabolic Syndrome, *M,F* Male, female, *QS* Quality Score, *OR* Odds Ratio, *CI* Confidence Interval, *IDF* International Diabetes Federation, *WHO* World Health Organization, *NCEP ATP III* National Cholesterol Education Program Adult Treatment Panel III, *LA* Los Angeles classification, *NR* Not Reported

### Meta-analysis of MetS and increased risk BE and sensitivity analysis

**Fig. 2:**
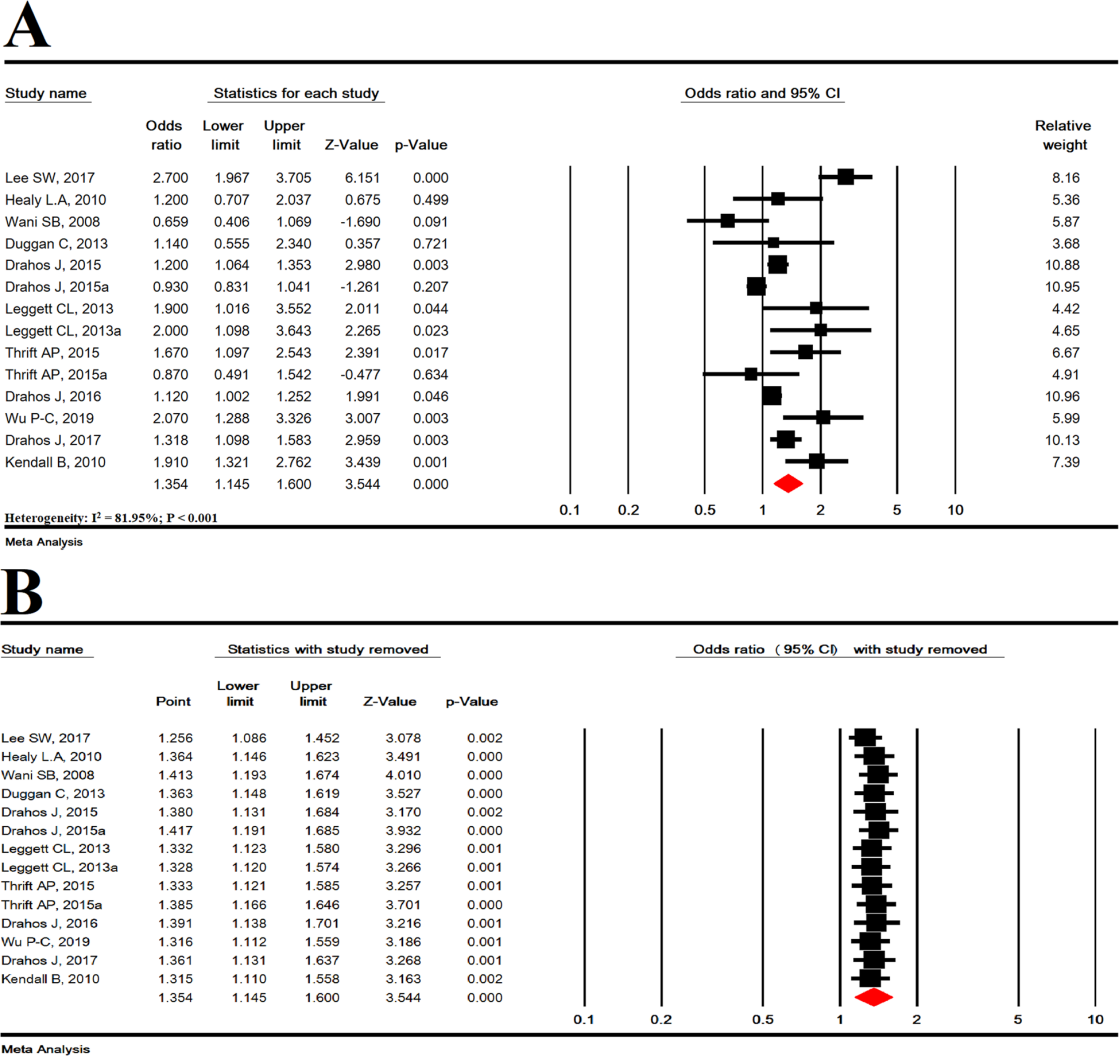
Meta-analysis (**a**) and sensitivity analysis (**b**) for the association between metabolic syndrome and increased risk of barrett’s esophagus

In 14 studies with a sample size of 108,416, MetS significantly increased the risk of BE (OR = 1.354; 95% CI: 1.145–1.600; *P* < 0.001; Heterogeneity: I^2^ = 81.95; *P* < 0.001) (Fig. 2a). Sensitivity analysis by omitting one study showed that overall estimates are still robust (Fig. 2b).

### MetS subgroup analysis and increased risk of BE

**Fig. 3:**
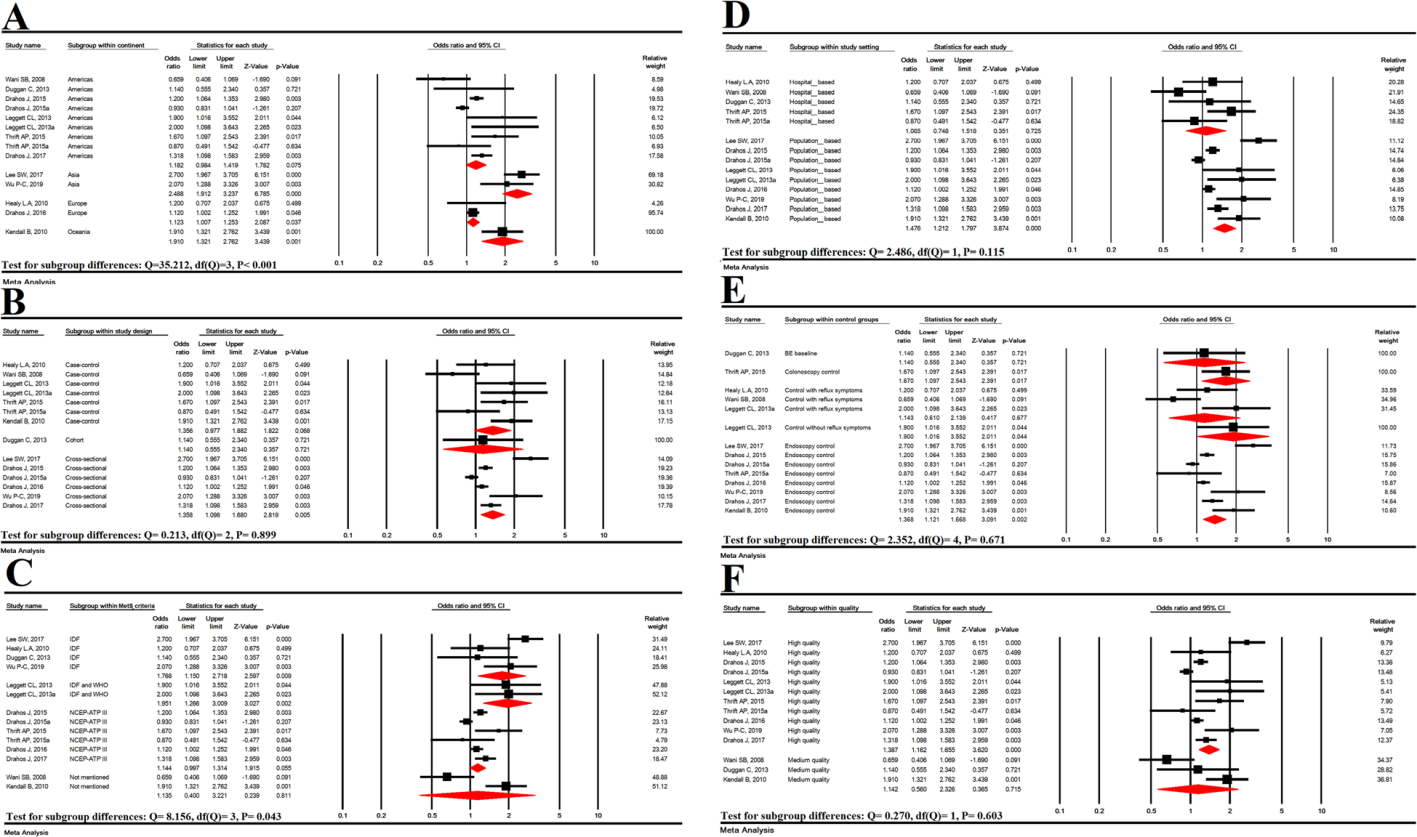
Subgroup analysis based on continents (**a**), study design (**b**), MetS diagnostic criteria (**c**), study setting (**d**) and control groups (**e**), study quality (**f**)

Subgroup analysis was significant for continent (*P* < 0.001) and MetS diagnostic criteria (*P* = 0.043), but was not significant for variables of study type (*P* = 0.899), study setting (*P* = 0.115), control groups (*P* = 0.671) and quality of studies (*P* = 0.603) (Fig. 3).

### Meta-regression and publication bias

**Fig. 4:**
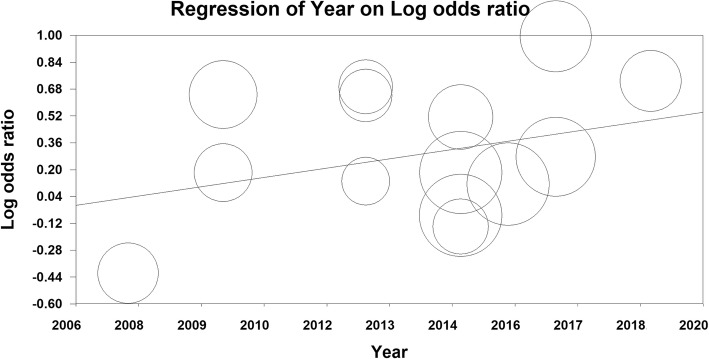
Meta-regression based on published year

The meta-regression model based on year of publication of articles was not significant for the relationship between MetS and BE (meta-regression coefficient: 0.041; 95% CI − 0.017 to 0.101; *P* = 0.167) (Fig. 4).

**Fig. 5:**
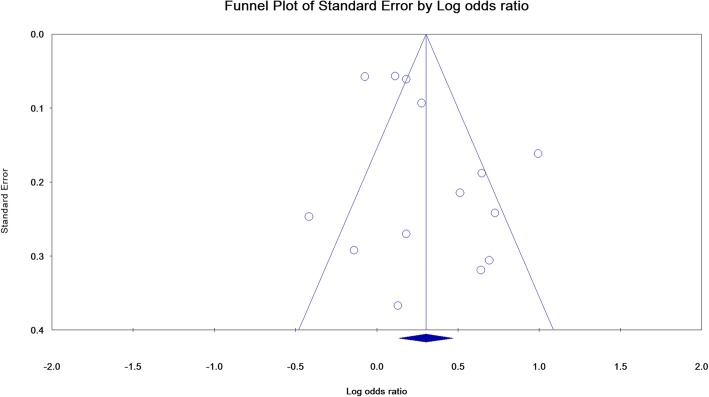
Publication bias

The publication bias is shown as a funnel plot, and the Begg (*P* = 0.912) and Egger’s (*P* = 0.094) tests were not significant; therefore, the publication bias did not play a role in the results (Fig. 5).

## Discussion

The present study is an update to the previous meta-analysis in 2016 [30], which found a significant relationship between MetS and BE (OR = 1.23; 95% CI: 1.03–1.47; *P* = 0.024) by combining eight studies. In the present study, a combination of 14 studies showed that MetS significantly increases the risk of BE (OR = 1.354; 95% CI: 1.145–1.600; *P* < 0.001). The strengths of the present study were the increase in the number of studies involved in meta-analysis and finding a more accurate relationship and a stronger level of significance for the relationship between MetS and BE. The causes of heterogeneity between the studies include the continent (*P* < 0.001) and MetS diagnostic criteria (*P* = 0.043).

In a systematic review and meta-analysis, age, male gender, smoking, longer BE segment, and low-grade dysplasia were risk factors for BE progression [31]. However, other studies continue to suggest that GERD is the strongest risk factor for BE. Moreover, the use of statin alone or in combination with aspirin as well as proton pump inhibitors (PPI) significantly reduced the risk of BE [31, 32]. In another meta-analysis, infection with Helicobacter pylori (*H. pylori*) also reduced the risk of BE [33].

BE is a precancerous condition for the EAC [2]. EAC is the most common type of esophageal cancer in the United States. Although GERD, smoking, and obesity have been suggested as associated risk factors, the major predictor of progression from non-dysplastic BE to EAC is the presence of dysplastic changes in esophageal histology [34]. Clinical guidelines recommend that periodic endoscopy be used to diagnose dysplasia and primary cancer in patients with BE, and this monitoring for BE patients may improve the prognosis of EAC [35].

A meta-analysis confirmed the conclusion that central adiposity can be strongly associated with esophageal inflammation and reflux [36]. Studies have shown that visceral obesity, as the main criterion for MetS, can increase the transient lower esophageal sphincter relaxation, the incidence of hiatal hernia, or even intra-abdominal pressure and acid reflux [37, 38].

In studies about hypertriglyceridemia, even after adjusting for obesity and other metabolic factors, it is associated with increased risk of BE [8]. Impairment of lipid metabolism is common in MetS. Abdominal obesity is a known risk factor associated with MetS. MetS is the result of obesity-related hormonal and systemic inflammatory changes and is associated with multi-system cancers in humans. There are several possible explanations for this relationship. First, insulin resistance and fatty liver may be responsible for elevated serum triglyceride (TG) levels, since fatty liver is significantly associated with fasting glucose and TG levels [39]. Hypertriglyceridemia is also associated with increased insulin resistance [40]. Second, since *H. pylori* infection is known to be a protective factor for erosive Esophagitis [41, 42] and chronic *H. pylori* infections can alter serum lipid profile, such as increasing total cholesterol and TG [43, 44], the increased serum TG levels can only be a side effect associated with *H. pylori* infection.

In other studies, the association between HTN and hypercholesterolemia (as MetS components) has also been demonstrated [45]. Atherosclerosis is recognized as an important factor for the development of HTN. In previous studies, atherosclerosis was associated with a high incidence of hiatal hernia. Loss of flexibility in the phrenoesophageal ligament in patients with arthrosclerosis and HTN is one of the causes of increased incidence of hiatal hernia [46].

In the present study, the meta-regression model based on year of publication of articles was not significant for the relationship between MetS and BE, which means that year of publication could not be a influencing factor on heterogeneity of studies.

This study has several strengths, including the fact that we used a comprehensive, concurrent search strategy to maximize the ability to identify all relevant literature. All stages of the research were conducted by two researchers independently, and the differences were resolved by discussion. We contacted the authors of the studies to obtain additional data. Based on the available data, we were able to identify some of the causes of heterogeneity between the studies.

One of the limitations of the present study is the high heterogeneity between the studies, though we attempted to discover the causes of heterogeneity through subgroup analysis. In addition, most studies were conducted in the United States, which may influence the results, according to continental analysis subgroup.

## Conclusion

MetS increases the risk of BE compared to control groups. The results of this study can help health practitioners by identifying a treatable risk factor for the most important risk factor for esophageal carcinoma (ie, BE). Future studies should examine whether treatment for MetS reduces the risk of BE and EA.

## Supplementary information

**Additional file 1.**

## Data Availability

Not applicable.

## Abbreviations

BE: Barrett’s esophagus
CI: Confidence interval
EAC: Esophageal adenocarcinoma
F: Female
GERD: Gastroesophageal reflux disease
H. pylori: Helicobacter pylori
HDL-C: High-density lipoprotein cholesterol
HTN: Hypertension
IDF: International Diabetes Federation
LA: Los Angeles classification
M: Male
MetS: Metabolic syndrome
MOOSE: Meta-analyses Of Observational Studies in Epidemiology
NCEP ATP III: National Cholesterol Education Program Adult Treatment Panel III
NR: Not Reported
OR: Odds ratio
PRISMA: Preferred Reporting Items for Systematic Reviews and Meta-analysis
QS: Quality Score
TG: Triglyceride
WHO: World Health Organization

## References

[1] Wang KK, Sampliner RE (2008). Practice parameters Committee of the American College of gastroenterology. Updated guidelines 2008 for the diagnosis, surveillance and therapy of Barrett's esophagus. Am J Gastroenterol.

[2] de Jonge PJ, van Blankenstein M, Grady WM, Kuipers EJ (2014). Barrett’s oesophagus: epidemiology, cancer risk and implications for management. Gut.

[3] van Soest EM, Dieleman JP, Siersema PD (2005). Increasing incidence of Barrett’s oesophagus in the general population. Gut.

[4] Shiota S, Singh S, Anshasi A, El-Serag HB (2015). Prevalence of Barrett’s esophagus in Asian countries: a systematic review and meta-analysis. Clin Gastroenterol Hepatol.

[5] Singh S, Sharma AN, Murad MH, Buttar NS, El-Serag HB, Katzka DA (2013). Central adiposity is associated with increased risk of esophageal inflammation, metaplasia, and adenocarcinoma: a systematic review and meta-analysis. Clin Gastroenterol Hepatol.

[6] Otaghi M, Azami M, Khorshidi A, Borji M, Tardeh Z (2019). The association between metabolic syndrome and polycystic ovary syndrome: a systematic review and meta-analysis. Diabetes Metab Syndr.

[7] Cooper-DeHoff RM, Pepine CJ (2007). Metabolic syndrome and cardiovascular disease: challenges and opportunities. Clin Cardiol.

[8] Ryan AM, Healy LA, Power DG, Byrne M, Murphy S, Byrne PJ (2008). Barrett esophagus: prevalence of central adiposity, metabolic syndrome, and a proinflammatory state. Ann Surg.

[9] Tan CE, Chew SK, Tai ES (2004). The metabolic syndrome: an Asian perspective. Inter Congr Series.

[10] Lee S-W, Lien H-C, Chang C-S, Lee T-Y, Peng Y-C, Yeh H-Z (2017). Association of metabolic syndrome with erosive esophagitis and Barrett’s esophagus in a Chinese population. J Chin Med Assoc.

[11] Healy LA, Ryan AM, Pidgeon G, Ravi N, Reynolds JV (2010). Lack of differential pattern in central adiposity and metabolic syndrome in Barrett's esophagus and gastroesophageal reflux disease. Dis Esophagus.

[12] Wani SB, Pondugula K, Bansal A, Rastogi A, Mathur S, Higbee A (2008). Metabolic syndrome is not a risk factor for Barrett's esophagus. Gastroenterology.

[13] Duggan C, Onstad L, Hardikar S, Blount PL, Reid BJ, Vaughan TL (2013). Association between markers of obesity and progression from Barrett’s esophagus to esophageal adenocarcinoma. Clin Gastroenterol Hepatol.

[14] Drahos J, Ricker W, Parsons R, Pfeiffer RM, Warren JL, Cook MB (2015). Metabolic syndrome increases risk of Barrett’s esophagus in the absence of gastroesophageal reflux: an analysis of SEER-Medicare data. J Clin Gastroenterol.

[15] Leggett CL, Nelsen EM, Tian J, Schleck CB, Zinsmeister AR, Dunagan KT (2013). Metabolic syndrome as a risk factor for Barrett esophagus: a population-based case-control study. Mayo Clin Proc.

[16] Thrift AP, Hilal J, El-Serag HB (2015). Metabolic syndrome and the risk of Barrett’s oesophagus in white males. Aliment Pharmacol Ther.

[17] Drahos J, Li L, Jick SS, Cook MB (2016). Metabolic syndrome in relation to Barrett's esophagus and esophageal adenocarcinoma: results from a large population-based case-control study in the clinical practice research Datalink. Cancer Epidemiol.

[18] Wu PC, Chen YH, Wu FZ, Lin KH, Hsu CL, Chen CS (2019). Risk factors for Barrett’s esophagus in young adults who underwent upper gastrointestinal endoscopy in a health examination center. Therap Adv Gastroenterol.

[19] Drahos J, Ricker W, Pfeiffer RM, Cook MB (2017). Metabolic syndrome and risk of esophageal adenocarcinoma in elderly patients in the United States: an analysis of SEER-Medicare data. Cancer.

[20] Kendall B, Macdonald G, Hayward N, Prins J, O'Brien S, Whiteman D (2010). Metabolic syndrome and the risk of Barrett’s oesophagus. J Gastroenterol Hepatol.

[21] Sayehmiri K, Tavan H. Systematic review and meta-analysis methods prevalence of peptic ulcer in IRAN. J Govaresh. 2015;20(4):250–8. 10.4103/jrms.JRMS_1035_16 PMC5813297 29456565

[22] Sayehmiri K, Tavan H, Sayehmire F, Mohamadi I. Prevalence of Epilepsy in iran using meta-analysis and systematic review. J Adv Med Biomed Res. 2015;23(97):112–21.

[23] Stroup DF, Berlin JA, Morton SC, Olkin I, Williamson GD, Rennie D, Moher D, Becker BJ, Sipe TA, Thacker SB (2000). Meta-analysis of observational studies in epidemiology: a proposal for reporting. Meta-analysis of observational studies in epidemiology (MOOSE) group. JAMA.

[24] Shamseer L, Moher D, Clarke M, Ghersi D, Liberati A, Petticrew M, PRISMA-P Group (2015). Preferred reporting items for systematic review and meta-analysis protocols (PRISMA-P) 2015:elaboration and explanation. BMJ.

[25] Wells GA, Shea B, O’Connell D, Peterson J, Welch V, Losos M, et al. The Newcastle-Ottawa Scale (NOS) for assessing the quality of nonrandomized studies in meta-analyses. 2011, [cited 2012 Nov 25]. Available from: http://www.ohri.ca/programs/clinical_epidemiology/oxford.asp.

[26] Higgins JPT, Green S (2011). Cochrane Handbook for Systematic Reviews of Interventions Version 5.1.0.

[27] Ades AE, Lu G, Higgins JP (2005). The interpretation of random-effects meta-analysis in decision models. Med Decis Mak.

[28] Begg CB, Mazumdar M (1994). Operating characteristics of a rank correlation test for publication bias. Biometrics.

[29] Egger M, Davey SG, Schneider M, Minder C (1997). Bias in meta-analysis detected by a simple, graphical test. BMJ.

[30] He Q, Li JD, Huang W, Zhu WC, Yang JQ (2016). Metabolic syndrome is associated with increased risk of Barrett esophagus: a meta-analysis. Medicine (Baltimore).

[31] Krishnamoorthi R, Singh S, Ragunathan K, Visrodia K, Wang KK, Katzka DA (2018). Factors associated with progression of Barrett’s esophagus: a systematic review and meta-analysis. Clin Gastroenterol Hepatol.

[32] Beales IL, Dearman L, Vardi I, Loke Y (2016). Reduced risk of Barrett’s esophagus in statin users: case-control study and meta-analysis. Dig Dis Sci.

[33] Erőss B, Farkas N, Vincze Á, Tinusz B, Szapáry L, Garami A (2018). Helicobacter pylori infection reduces the risk of Barrett’s esophagus: a meta-analysis and systematic review. Helicobacter.

[34] Runge TM, Abrams JA, Shaheen NJ (2015). Epidemiology of Barrett’s esophagus and esophageal adenocarcinoma. Gastroenterol Clin N Am.

[35] Ding YE, Li Y, He XK, Sun LM (2018). Impact of Barrett’s esophagus surveillance on the prognosis of esophageal adenocarcinoma: a meta-analysis. J Dig Dis.

[36] Singh S, Sharma AN, Murad MH, Buttar NS, El-Serag HB, Katzka DA (2013). Central adiposity is associated with increased risk of esophageal inflammation, metaplasia, and adenocarcinoma: a systematic review and meta-analysis. Clin Gastroenterol Hepatol.

[37] Barak N, Ehrenpreis ED, Harrison JR, Sitrin MD (2002). Gastro-oesophageal reflux disease in obesity: pathophysiological and therapeutic considerations. Obes Rev.

[38] Wu P, Ma L, Dai GX, Chen Y, Tong YL, Wang C (2011). The association of metabolic syndrome with reflux esophagitis: a case-control study. Neurogastroenterol Motil.

[39] Long E, Beales IL (2014). The role of obesity in oesophageal cancer development. Ther Adv Gastroenterol.

[40] Nguyen-Duy TB, Nichaman MZ, Church TS, Blair SN, Ross R (2003). Visceral fat and liver fat are independent predictors of metabolic risk factors in men. Am J Physiol Endocrinol Metab.

[41] Moro E, Gallina P, Pais M, Cazzolato G, Alessandrini P, Bittolo-Bon G (2003). Hypertriglyceridemia is associated with increased insulin resistance in subjects with normal glucose tolerance: evaluation in a large cohort of subjects assessed with the 1999 World Health Organization criteria for the classification of diabetes. Metabolism.

[42] Labenz J, Blum AL, Bayerdorffer E, Meining A, Stolte M, Borsch G (1997). Curing helicobacter pylori infection in patients with duodenal ulcer may provoke reflux esophagitis. Gastroenterology.

[43] Tsukada K, Katoh H, Miyazaki T, Fukuchi M, Kuwano H, Kimura H, Fukai Y, Inose T, Motojima T, Toda N, Yamada S (2006). Factors associated with the development of reflux esophagitis after helicobacter pylori eradication. Dig Dis Sci.

[44] Laurila A, Bloigu A, Nayha S, Hassi J, Leinonen M, Saikku P (1999). Association of Helicobacter pylori infection with elevated serum lipids. Atherosclerosis.

[45] Gudlaugsdottir S, Verschuren W, Dees J, Stijnen T, Wilson J (2002). Hypertension is frequently present in patients with reflux esophagitis or Barrett’s esophagus but not in those with non-ulcer dyspepsia. Eur J Intern Med.

[46] Furuta K, Adachi K, Arima N, Yagi J, Tanaka S, Miyaoka Y (2007). Study of arteriosclerosis in patients with hiatal hernia and reflux esophagitis. J Gastroenterol Hepatol.

